# Assessing Surgical Feasibility of Peritoneal Dialysis Following Liver–Lung Transplantation: A Case Report

**DOI:** 10.1155/crit/5753967

**Published:** 2026-08-19

**Authors:** Charlotte Cornwell, Margaret Golding, Erin Vaughan, Jerome Laurence, Susanna Lam

**Affiliations:** ^1^ The University of Sydney, Camperdown, New South Wales, Australia, sydney.edu.au; ^2^ Transplantation Services, Royal Prince Alfred Hospital, Camperdown, New South Wales, Australia, nsw.gov.au

**Keywords:** liver transplant, lung transplant, peritoneal dialysis

## Abstract

Kidney failure is a common complication in recipients of nonkidney solid organ transplants (NKSOT), often stemming from prolonged calcineurin inhibitor (CNI) exposure or perioperative insults. Although haemodialysis (HD) remains the predominant renal replacement therapy (RRT), peritoneal dialysis (PD) offers advantages such as better quality of life, haemodynamic stability and preservation of residual renal function. Concerns about infection risk, technical feasibility and respiratory complications have curtailed its broader use. We present the first reported case of successful PD in a liver and double‐lung transplant recipient. The patient, a 30‐year‐old male with cystic fibrosis, extensive abdominal surgery and immunosuppression, was initially deemed unsuitable for PD due to surgical complexity and potential complications in a transplant recipient. A multidisciplinary team (MDT) review at our liver and kidney transplant specialist centre ultimately supported PD based on clinical suitability and lifestyle preferences. Laparoscopic catheter insertion was achieved despite significant intra‐abdominal adhesions, and the patient remained on PD for 18 months without complications. This case underscores that with meticulous surgical planning and MDT input, PD can be a safe and viable option in complex NKSOT recipients. It challenges prevailing biases against PD in this population and suggests that prior transplantation should not preclude its consideration.

## 1. Introduction

Kidney failure is common following transplantation, often secondary to nephrotoxicity from immunosuppressive therapies or physiological insults in the perioperative period [1, 2]. In recipients of nonkidney solid organ transplants (NKSOT) requiring renal replacement therapy (RRT), haemodialysis (HD) has traditionally been the preferred modality over peritoneal dialysis (PD). This preference stems from concerns for an increased risk of peritonitis, technical challenges related to peritoneal catheter placement in patients with prior major abdominal surgery, and the potential for pulmonary complications in lung transplant patients [3–6]. Although successful use of PD has been reported in patients with previous lung, heart or liver transplants, its role and safety in individuals who have undergone both lung and liver transplantation remain unclear. This is the first reported case of a patient with kidney failure successfully established on PD, on a background of a liver and subsequent double lung transplant.

## 2. Case Report

We present the case of a 30‐year‐old male with cystic fibrosis (CF) who underwent both liver and lung transplantation and was successfully established on PD after developing kidney failure from prolonged calcineurin inhibitor (CNI) use. To our knowledge, no other cases of successful PD in patients who have undergone both liver and lung transplantation have been reported.

The patient received a deceased donor whole paediatric liver graft at age 10 via a Mercedes Benz incision, with Roux‐en‐Y hepaticojejunostomy reconstruction. On postoperative Day 6, he required a relook laparotomy due to major abdominal haemorrhage. Twelve years later, a laparotomy was performed for closure of a percutaneous endoscopic gastrostomy‐related gastroenteric fistula. The patient also had a history of laparoscopic appendicectomy. There were no further gastrointestinal complications, and his liver allograft function remained adequate despite chronic rejection. Fourteen years after the liver transplant, the patient underwent a double lung transplant via clamshell thoracotomy, achieving good respiratory function. His past medical history was also notable for hypertension and osteopenia. His current medications included vitamin D, magnesium, calcium, tacrolimus, prednisone, posaconazole and pancrelipase.

Over time, however, due to prolonged CNI therapy and CF–related diabetes, he developed progressive renal failure. This deterioration accelerated more recently, with his eGFR declining to 7 mL/min and serum creatinine rising to 840 *μ*mol/L. A renal biopsy revealed extensive chronic changes with evidence of chronic thrombotic microangiopathy. He consequently required expedited access for RRT.

He was evaluated by renal and surgical teams at a regional hospital and considered unsuitable for PD; however HD access was recommended instead. The patient expressed significant concerns about how HD could affect his quality of life and ability to continue working full‐time. Consequently, his hepatologist and transplant respiratory physician referred him to our quaternary‐care centre, Royal Prince Alfred Hospital, Sydney, in September 2023, for a second opinion on the technical feasibility of laparoscopic PD catheter insertion and overall suitability for PD, given his extensive abdominal surgical history and dual‐organ transplant background.

The patient was reviewed in the dialysis access clinic by the nephrology team, transplant surgery, the CKD clinical nurse consultant (CNC) and the PD CNC. On examination, he appeared well. His weight was 59 kg, blood pressure 150/110 mmHg and heart rate 80 bpm. He had a bilateral clamshell thoracotomy scar, with reduced air entry bilaterally but no crackles. There was no peripheral oedema. Abdominal examination revealed a Mercedes Benz incision and a small, reducible umbilical hernia, with no evidence of groin hernias.

The patient was then discussed at an MDT meeting with transplantation surgery, hepatology, nephrology, respiratory and endocrinology teams. The risks of PD, including volume overload, peritonitis and exit site infections, were discussed. Additionally, specific risks for PD catheter insertion in transplant patients, such as pleural‐peritoneal fistula, pleural effusion, poor wound healing due to immunosuppression and reduced lung function due to increased intra‐abdominal pressure, were addressed. In discussion with nephrology, hepatology and lung transplant teams, immunosuppression was switched from everolimus to tacrolimus 4 weeks prior to surgery to assist wound healing and was briefly withheld perioperatively. Despite these risks, the patient preferred PD due to his full‐time employment, young family and distance to a HD centre. After careful evaluation, the MDT unanimously agreed to proceed with the PD catheter insertion. He received education on PD with the PD CNC and preoperative washing instructions.

The patient was admitted for laparoscopy and PD catheter insertion. The preoperative period was complicated by hyperkalaemia, parainfluenza infection and carbapenemase‐producing Enterobacterales colonisation, which were medically managed. Under general anaesthesia, a 5‐mm infraumbilical Hasson port was inserted to establish pneumoperitoneum, followed by a 5‐mm right lower quadrant port. The abdomen showed extensive adhesions above the liver and spleen, but the pelvis was free of adhesions. A 16 Fr coiled PD catheter trocar was placed left of midline and tunnelled in the retrorectus space and through the peritoneum above the bladder under vision. Initially, a 57 cm 15 Fr coiled PD catheter from a PD kit was inserted under vision via the trocar above the pelvis, but it was exchanged for a 62‐cm catheter, to allow the curl of the catheter to be positioned in the pelvis in the rectovesical pouch. The catheter was tunnelled through subcutaneous tissue to the left, with the internal cuff at the fascia and the external cuff under the skin. After instilling and draining 500 mL of fluid, good flow was confirmed. The ports were removed, and the wounds were closed with 3‐0 Vicryl and 3‐0 Monocryl and dressed with sterile waterproof dressing, which remained intact for 2 weeks. He was given further education and advised to not shower until review by the PD CNC team. The patient recovered well and was discharged on postoperative day one. He was reviewed in the dialysis access MDT clinic 2 weeks postoperatively, where the PD site was clean and laparoscopic scars healing well. His care was transferred back to his regional centre where PD training commenced successfully. The patient remained on PD for 18 months with no complications and subsequently received a deceased donor kidney transplant in March 2026. The key clinical events are summarised chronologically in Table 1.

**Table 1:** Timeline of clinical events.

Date	Clinical event
2005	Liver transplant
2019	Lung transplant
September 2023	Referred to quatarenery transplant centre, multidisciplinary assessment for feasibility of peritoneal dialysis
October 2023	Peritoneal dialysis catheter inserted
November 2023	Commencement of peritoneal dialysis
December 2023–February 2026	Follow‐up demonstrating successful peritoneal dialysis without any respiratory compromise or transplant‐related complications
March 2026	Deceased donor kidney transplant

## 3. Discussion

Kidney disease is a common complication amongst recipients of NKSOT and carries significant implications for long‐term morbidity and mortality. Its aetiology is multifactorial, encompassing preoperative, perioperative and postoperative insults. These may include shared risk factors across transplant types, as well as mechanisms unique to specific organs. In liver transplant recipients, the incidence of acute kidney injury (AKI) ranges from 17% to 95%, with 5%–35% of those affected requiring RRT [1]. Amongst lung transplant recipients, AKI has been reported in 5%–60% of cases, with 8%–10% progressing to require RRT [1]. Many patients who have undergone liver or lung transplantation, and those who have received both, are managed with HD. PD is less commonly utilised, largely due to perceived risks such as peritonitis, catheter‐related complications and technical challenges related to catheter insertion. However, evidence supporting these concerns remains limited [2, 6].

PD offers several important advantages over HD, making it a preferable option for many patients with kidney failure. PD provides better survival rates, especially in the first year of treatment, and carries a lower risk of infection and all‐cause mortality compared with HD. PD′s gentle, continuous removal of toxins reduces haemodynamic instability, making it suitable for critically ill and frail patients. Additionally, PD preserves residual kidney function better than HD, improves quality of life by offering more autonomy and flexibility, and avoids the discomfort of repeated vascular access. It can be done at home, even during sleep and has significant economic benefits due to lower resource demands. Moreover, pretransplant PD is associated with better posttransplant outcomes. These factors support the growing preference for PD as an effective and safe dialysis modality [7].

PD catheter insertion can be technically challenging in patients who have undergone combined lung and liver transplantation. Prior abdominal surgeries and the cumulative surgical trauma associated with dual‐organ transplantation can significantly increase technical complexity and perioperative risk, making meticulous preoperative planning and the involvement of experienced surgical teams skilled in advanced laparoscopic techniques essential for optimising outcomes. Liver transplantation requires extensive manipulation of the peritoneal cavity, including hepatic mobilisation and perihepatic dissection, which can result in adhesion formation and altered intra‐abdominal anatomy. Additionally, the placement of surgical drains during transplantation may contribute to chronic fluid loculations, further complicating subsequent catheter placement. Despite these anatomical and surgical challenges, successful PD catheter insertion has been documented in NKSOT recipients, particularly when performed laparoscopically. Laparoscopic techniques offer the advantage of direct visualisation and allow for adhesiolysis, thereby improving catheter function and placement accuracy. A history of abdominal surgery does not appear to negatively impact PD catheter outcomes and, in most cases, should not be considered a contraindication to PD [8]. More recent studies have reinforced these findings, demonstrating that advances in laparoscopic insertion techniques and specialised multidisciplinary care have improved PD catheter outcomes in patients with previous abdominal surgery, supporting broader consideration of PD in carefully selected patients [5, 9]. Abandonment of the catheter insertion procedure or discontinuation of PD due to catheter failure in these patients is rare [5, 9].

Several studies have examined whether patients on PD with a history of liver transplantation face an increased risk of infectious complications, including peritonitis and exit site infections. These studies have consistently demonstrated that the risk of peritonitis in this population is comparable to that observed in PD patients that are immunosuppressed but with no history of liver transplantation [3, 10]. Additionally, episodes of peritonitis in liver transplant recipients are not associated with higher rates of complications such as technique failure, graft dysfunction or mortality [3, 11].

Patients undergoing PD can experience a range of respiratory complications due to the physical and metabolic effects of the dialysis process [4]. The infusion of dialysate into the abdominal cavity raises intra‐abdominal pressure, which can reduce lung volumes by elevating the diaphragm and limiting lung expansion, especially in patients with compromised lung function or lung transplant recipients. Small but significant reductions in vital capacity, lung compliance and oxygen exchange occur following dialysate infusion, which can cause dyspnoea or hypoxia [12]. Additionally, larger dialysate volumes are associated with greater pulmonary function impairment, and excessive intra‐abdominal pressure may rarely lead to complications such as hydrothorax. PD may also affect ventilation through increased glucose absorption, leading to hyperventilation or, in rare cases, respiratory acidosis. Although these effects may lessen over time, the respiratory burden of PD should be carefully considered in lung transplant recipients, where maintaining optimal pulmonary mechanics and gas exchange is critical. Tailoring PD protocols, such as limiting fill volumes or adjusting patient positioning, can help reduce respiratory strain in this population [13]. In our patient, PD was well tolerated without respiratory compromise, and no specific interventions were required. Furthermore, the patient tolerated a standard PD prescription without the need for significant individualisation.

## 4. Conclusion

From a surgical perspective, PD is a safe and viable RRT for patients who have undergone both liver and lung transplantation. When supported by careful patient selection, multidisciplinary assessment and meticulous surgical technique, PD can be successfully and safely implemented in this complex population. Although overall survival rates are comparable between transplant recipients on HD and those on PD, patients managed with PD may experience lower rates of hospitalisation. Catheter‐related complications, including peritonitis, occur at rates similar to nontransplant PD populations, with no evidence of heightened risk in carefully managed cases. This case highlights the critical importance of early referral to an experienced dialysis‐access MDT to assess technical feasibility of laparoscopic catheter insertion for PD. Equally essential is close communication with transplant physicians for preoperative optimisation and to minimise potential complications. Despite evidence supporting its safety and efficacy, PD remains underutilised in this cohort, likely due to perceived rather than actual risks. With appropriate planning and collaborative care, PD should be considered a legitimate and effective option for these patients. Although our case supports the feasibility of PD following combined liver‐lung transplantation, larger‐scale observational studies are required to better characterise outcomes, safety and optimal patient selection.

## Author Contributions

Charlotte Cornwell (unaccredited surgical registrar): conception, collection and interpretation of clinical data, literature review, preparation of the timeline of events, draught and revision of manuscript.

Margaret Golding (dialysis access clinical nurse consultant): contribution to patient care and provision of expertise regarding dialysis management, review and approval of final manuscript.

Erin Vaughan (renal transplant physician): contribution to clinical management of patient, provision of specialist nephrology input, critical review of the manuscript and approval of final version.

Jerome Laurence and Susanna Lam (transplant surgeons): draught and revision of manuscript, contribution to patient′s transplant care and management, critical review of the manuscript and approval of the final version for publication.

## Funding

No funding was received for this manuscript.

## Disclosure

All authors read and approved the final manuscript.

## Consent

Written informed consent was obtained from the patient for publication of this case report. A copy of the written consent is available for review by the editor upon request.

## Conflicts of Interest

The authors declare no conflicts of interest.

## Data Availability

Data sharing is not applicable to this article as no datasets were generated or analysed during the current study.
